# Association between Healthy Eating Index-2015 total and metabolic associated fatty liver disease in Americans: a cross-sectional study with U.S. National Health and Nutrition Examination Survey

**DOI:** 10.3389/fnut.2024.1427619

**Published:** 2025-01-13

**Authors:** Genzhong Xu, Ermin Ma, Weitao Zhang, Bo Feng

**Affiliations:** The First Affiliated Hospital of Henan University of CM, Zhengzhou, Henan, China

**Keywords:** Healthy Eating Index, metabolic associated fatty liver disease, NHANES, dietary quality, cross-sectional survey

## Abstract

**Background:**

Utilizing the National Health and Nutrition Examination Survey (NHANES) dataset to investigate the relationship between dietary quality, as assessed by the Healthy Eating Index-2015 (HEI-2015), and the prevalence of metabolic associated fatty liver disease (MAFLD) among adults in the United States, our analysis revealed that an increased dietary quality was significantly correlated with a reduced risk of MAFLD in the American population.

**Method:**

The NHANES dataset, encompassing the years 2017–2018 and comprising 3,557 participants, was incorporated into our analytical framework. Weighted multivariate linear regression model was performed to assess the linear relationship between the HEI-2015 and MAFLD. Dietary intake data were derived from two 24-h dietary recall interviews conducted as part of NHANES.

**Results:**

Following multivariable adjustment, the weighted multivariable linear regression models demonstrated a negative correlation between the HEI-2015 total scores and the risk of MAFLD. The weighted logistic regression models revealed that each unit of increased HEI-2015 total value was associated with a 1.2% (95% CI: 0.9%, 1.5%; *P* < 0.001) decrease in the risk of f MAFLD. Upon categorization of the HEI-2015 scores into quartiles, the odds ratios (ORs) for the association between the risk of MAFLD and the quartile scores of HEI-2015, in comparison to the baseline quartile, were 0.945 (95% CI: 0.852–1.047; *P* = 0.279), 0.834 (95% CI: 0.750–0.927; *P* < 0.001), and 0.723 (95% CI: 0.646–0.811; *P* < 0.001), respectively. When participants were stratified by age and sex, subgroup analyses showed a similar trend. This pattern was also evident in the smooth curve fitting (SCF) and weighted generalized additive model (GAM).

**Conclusion:**

Elevated dietary quality, as assessed by the total and component food scores of the HEI-2015, was significantly correlated with a diminished risk of MAFLD among participants in the NHANES survey featured in this investigation.

## 1 Background

Metabolic Associated Fatty Liver Disease (MAFLD) has emerged as a redefined condition that supersedes the previous classification of non-alcoholic fatty liver disease (NAFLD), with a diagnostic focus on metabolic dysfunction rather than the exclusion of other liver diseases (1). The prevalence of MAFLD is escalating, imposing a substantial clinical and economic burden on society, and currently, there is no approved pharmacological treatment for this condition (2). The progression of MAFLD can be influenced by various factors, including lifestyle modifications (3), co-existing conditions such as type 2 diabetes mellitus (4), genetic predisposition (5), and in some cases, it may even experience spontaneous regression (1). Evidence from several studies suggests that dietary changes can prevent the onset of MAFLD, reduce liver fat in those already affected, and safeguard cardiovascular health (6, 7).

The Healthy Eating Index 2015 (HEI-2015) is a comprehensive tool used to assess dietary quality, aligning with the Dietary Guidelines for Americans (DGA) (8, 9). Unlike measures focused on absolute dietary intake, the HEI-2015 evaluates components on a density basis (e.g., per 1,000 kcal), providing a more nuanced view of dietary habits. This index comprises 13 distinct component scores, and their collective analysis helps to unravel the intricacies of dietary patterns and their interplay (8). Composite indices like the HEI-2015, which reflect overall dietary quality, have been instrumental in identifying and assessing the risks associated with diet-related metabolic disorders, including metabolic syndrome (MetS) (10) and ulcerative colitis (11), among others.

In our study, we aim to investigate the significant association between higher dietary quality, as measured by the HEI-2015, and the reduced risk of developing MAFLD. By examining this relationship, we hope to offer evidence-based dietary recommendations for the prevention and management of MAFLD, contributing to the broader understanding of lifestyle interventions in metabolic health.

## 2 Subjects and methods

### 2.1 Study population

The present study used data from the National Health and Nutrition Examination Survey (NHANES) (https://www.cdc.gov/nchs/nhanes/index.htm). NHANES is a cross-sectional survey conducted by the National Center for Health Statistics (NCHS) of the Centers for Disease Control and Prevention (CDC) to assess the prevalence of diseases and their risk factors in the US population (12). This study was drawn from the most recent wave of NHANES 2017–2018, which assessed liver function using ultrasound and vibration-controlled transient elastography (VCTE) for the first time in the survey. A median Controlled Attenuation Parameter (CAP) defined the liver steatosis and a median Liver Stiffness Measurement (LSM) defined the liver fibrosis.

The selection criteria were as follows: (1) Completion of the elastography exam; (2) Availability of dietary information; (3) Age of participants above 20 years old.

**Figure 1:**
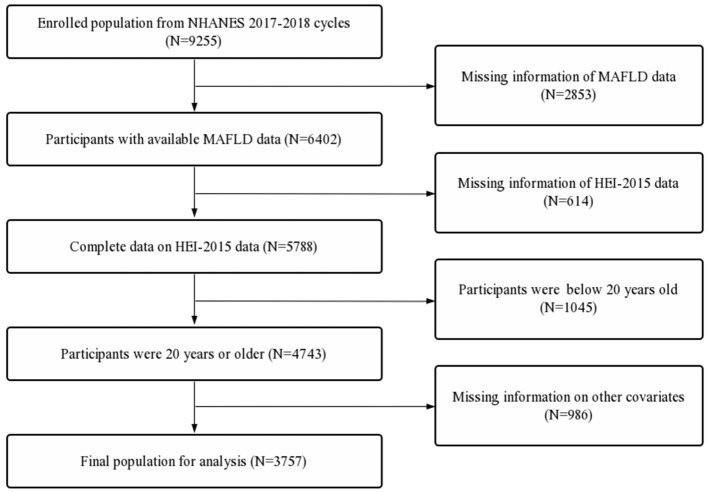
Flowchart of study participates.

The exclusion criteria were as follows: (1) Participants with incomplete data on race/ethnicity, body mass index (BMI), poverty income ratio (PIR), educational level, marital status, diabetes status, hdypertension status, drinking status, smoking status and activity status. (2) Incomplete lab panel with eGFR, estimated glomerular filtration rate; ALT, alanine transaminase; AST, aspartate transaminase; albumin; globulin; ALP, alkaline phosphatase; LDH, lactate dehydrogenase; total bilirubin; total protein; uric acid; HbA1c, glycated hemoglobin; HDL, high density lipoprotein; LDL, low density lipoprotein; triglycerides; hsCRP, high sensitivity C-reactive protein (Figure 1).

This survey was approved by the National Center for Health Statistics Research Ethics Review Board (Protocol number: 2018-01) and written informed consent was obtained from all participants.

### 2.2 Diagnosis of MAFLD

MAFLD is identified through the presence of hepatic steatosis coupled with one or more of the following criteria: overweight, diabetes mellitus, or metabolic dysfunction (13). Overweight/obesity is defined as a BMI ≥ 25. Type 2 diabetes is classified into two categories: normal (HbA1c levels below 5.7% and no reported diabetes) and diabetic (HbA1c levels at or above 6.5% or reported diabetes). Metabolic dysfunction, as per MAFLD criteria, is characterized by the fullfillment of at least two of the following conditions: (1) an enlarged waist circumference (over 102 cm for men and over 88 cm for women), (2) high blood pressure (systolic/diastolic readings above 130/85 mmHg or on medication for hypertension), (3) elevated triglyceride levels (over 1.70 mmol/L or under pharmacological treatment), (4) reduced high-density lipoprotein cholesterol (HDL-C below 1.0 mmol/L for men and below 1.3 mmol/L for women), (5) prediabetes, (6) elevated highly sensitive C-reactive protein levels (above 2 mg/L).

The degree of hepatic steatosis in the liver is evaluated using ultrasound and vibration-controlled transient elastography (VCTE), with the Controlled Attenuation Parameter (CAP) score indicating its presence. CAP scores vary from 100 to 400 dB/m, where higher scores suggest greater hepatic fat content. Steatosis is graded from S0 to S3, with CAP thresholds at 248, 268, and 280 for grades S1, S2, and S3, respectively. In our study, a CAP score of 248 dB/m or higher is considered to represent a steatosis level beyond S0.

### 2.3 Assessment of dietary quality

The overall diet quality was characterized using the Healthy Eating Index (HEI) recommended by the US Department of Agriculture (USDA) (14). The HEI-2015 includes 13 components, including 9 adequacy components (total vegetables, greens and beans, total fruits, whole fruits, whole grains, dairy, total protein foods, seafood and plant proteins, and fatty acids) and 4 moderation components (sodium, refined grains, saturated fats, and added sugars) (8, 9). Dietary intake data were obtained from two NHANES 24-h recall interviews, which were used to calculate dietary scores from the Food Patterns Equivalents Database files. The first interview was conducted in person at the Mobile Examination Center (MEC), and the second by telephone 3–10 days later. Dietary intake was estimated using the mean of the two 24-h recall data.

### 2.4 Description and analysis of covariates

Demographic characteristics presented on the NHANES website as potential confounders were considered covariates in our study analyses and included age (20–55 years, ≥55 years), gender (male, female), race/ethnicity (Mexican Americans, other Hispanic, non-Hispanic White, non-Hispanic Black, other race), BMI (< 25, 25–30, ≥30), educational level (< 9th grade, 9–11th grade, high school, some college, college graduate), marital status (married, widowed, divorced, separated, never married, living with partner), and income status classified into three levels of the poverty income ratio (PIR; ≤ 1, 1–3, >3), which was calculated by dividing family (or individual) income by the poverty guidelines specific to the survey year. Based on a review of the literature and our clinical experience, other covariate data were obtained from the corresponding questionnaire, including diabetes status (yes or no), hypertension status (yes or no), smoked at least 100 cigarettes (yes or no), alcohol consumption (drink/day), and moderate or vigorous activity (yes or no).

### 2.5 Statistical analysis

According to the weight selection criteria of NHANES, sampling weights were used in all analyses. The mean and proportion were used to describe data from continuous and categorical variables, respectively. Chi-square test was used to compare the differences of categorical variables between the MAFLD and non-MAFLD groups, and for continuous variables, a Student's *t*-test was used. Weighted multivariate linear regression model was performed to assess the linear relationship between the HEI-2015 and MAFLD. The odds ratios and 95% confidence intervals (95% CIs) for the association between the HEI-2015 and the risk of MAFLD were assessed using a weighted logistic regression model. Model 1 was adjusted for no covariates. Model 2 was adjusted for age (if applicable), sex (if applicable), and race. Model 3 was adjusted for all the applicable covariates. Subgroup analyses based on sex and age were further performed via weighted stratified line regression models. Moreover, the nonlinear relationship was characterized by smooth curve fitting (SCF) and weighted generalized additive model (GAM). Furthermore, the following analyses were performed to ensure the robustness of the data analysis. The values of HEI-2015 were categorized based on quartiles, and tests for linear trends were performed. All the steps described above were also performed to evaluate the relationship between the categorized HEI-2015 and the risk of MAFLD. All analyses were performed via R software (4.0.3) and Empower Stats (2.0). A two-sided *P* < 0.05 was considered to have statistical significance.

## 3 Results

### 3.1 Baseline characteristics of participants

First, a total of 9,255 participants were extracted. Second, participants with missing MAFLD data (*n* = 2,853) and incomplete HEI-2015 data (*n* = 614) were excluded. Further, participants below 20 years old (*n* = 1,054) and participants with missing data on other covariates (*n* = 986) were also excluded. A total of 3,757 participants were included in the final analysis (Figure 1).

Compared with the non-MAFLD group, participants in the MAFLD group tended to have less total vegetables (3.1 ± 1.7 vs. 2.8 ± 1.7, *P* < 0.001), greens and beans (1.7 ± 2.2 vs. 1.3 ± 2.0, *P* < 0.001), and total fruits (1.9 ± 2.0 vs. 1.7 ± 2.0, *P* < 0.050), whole fruits (2.1 ± 2.3 vs. 1.8 ± 2.2, *P* < 0.050), fatty acids (5.0 ± 3.6 vs. 4.6 ± 3.7, *P* < 0.050) and refined grains (6.4 ± 3.7 vs. 5.9 ± 3.7, *P* < 0.050) intake. In addition, the percentage of participants who had a higher hypertension, and diabetes were significantly higher in the MAFLD group. Participants in the MAFLD group were more likely to be older, male, fatter, married, have less activity, and have lower educational levels (*P* < 0.050, Table 1).

**Table 1:** Weighted characteristics of the study population.

	**Non-MAFLD (*N* = 2,075, 55.2%)**	**MAFLD (*N* = 1,682, 44.8%)**	***P* value**
**Age (%)**			
< 55	64.4	50.5	< 0.0001
≥55	35.6	49.5	
**Sex (%)**			
Male	43.3	53.1	< 0.0001
Female	56.7	46.9	
**Race (%)**			
Mexican Americans	5.8	10.7	< 0.0001
Other Hispanic	6.8	6.1	
Non-Hispanic White	65.6	65.3	
Non-Hispanic Black	12.2	9.0	
Other race	9.6	8.8	
**BMI (%)**			
< 25	42.1	3.9	< 0.0001
≥25, < 30	31.8	27.7	
≥30	26.1	68.4	
**PIR (%)**			
< 1	10.5	10.4	0.5466
≥1, < 3	31.2	33.4	
≥3	49.5	47.6	
NA	8.8	8.6	
**Educational level (%)**			
< 9th grade	2.6	3.0	< 0.0001
9–11th grade	6.9	6.8	
High school	25.6	28.3	
Some college	29.0	33.5	
College graduate	36.0	28.3	
**Marital status (%)**			
Married	50.7	60.2	< 0.0001
Widowed	4.6	6.3	
Divorced	11.6	9.2	
Separated	2.5	2.5	
never married	21.5	13.0	
Living with partner	9.1	8.8	
**Diabetes status (%)**			
Yes	7.3	27.2	< 0.0001
No	87.0	62.0	
Borderline	5.7	10.9	
**Hypertension status (%)**			
Yes	23.6	48.3	< 0.0001
No	76.4	51.7	
**eGFR [mL/(min**−**1.73 m**^2^**), %]**			
< 60	6.5	8.3	0.0019
≥60, < 90	32.2	35.9	
≥90	61.3	55.8	
**Smoked at least 100 cigarettes (%)**			
Yes	40.4	44.3	0.0178
No	59.6	55.7	
Alcohol consumption (drink/day, mean ± SD)	1.3 ± 3.2	1.3 ± 3.0	0.4580
**Moderate or vigorous activity (%)**			
Yes	61.1	48.4	< 0.0001
No	38.9	51.6	
**Lab panel**			
ALT (IU/L, mean ± SD)	19.5 ± 13.5	27.3 ± 19.3	< 0.0001
AST (IU/L, mean ± SD)	21.4 ± 13.7	23.3 ± 12.5	< 0.0001
AST/ALT (mean ± SD)	1.2 ± 0.4	1.0 ± 0.3	< 0.0001
Albumin (g/dl, mean ± SD)	4.1 ± 0.3	4.1 ± 0.3	0.0018
Globulin (g/dl, mean ± SD)	3.0 ± 0.4	3.0 ± 0.4	< 0.0001
Albumin/globulin (mean ± SD)	1.4 ± 0.2	1.4 ± 0.2	< 0.0001
GGT (U/L, mean ± SD)	24.7 ± 39.9	35.8 ± 38.9	< 0.0001
ALP (IU/L, mean ± SD)	74.0 ± 27.6	80.3 ± 22.8	< 0.0001
LDH (U/L, mean ± SD)	156.2 ± 31.7	159.1 ± 32.6	0.0051
Total bilirubin (mg/dl, mean ± SD)	0.5 ± 0.3	0.5 ± 0.3	0.0022
Total protein (g/dl, mean ± SD)	7.1 ± 0.4	7.1 ± 0.4	0.0364
Uric acid (mg/dl, mean ± SD)	5.1 ± 1.3	5.8 ± 1.4	< 0.0001
HbA1c (%, mean ± SD)	5.5 ± 0.6	6.0 ± 1.1	< 0.0001
HDL (mg/dl, mean ± SD)	57.6 ± 15.9	48.6 ± 13.3	< 0.0001
Total cholesterol (mg/dl, mean ± SD)	187.5 ± 39.9	192.6 ± 41.7	0.0002
LDL (mg/dl, mean ± SD)	108.9 ± 33.8	113.3 ± 38.2	0.0087
Triglycerides (mg/dl, mean ± SD)	114.8 ± 65.2	174.0 ± 106.6	< 0.0001
hsCRP (mg/L, mean ± SD)	3.1 ± 6.3	5.0 ± 8.7	< 0.0001
**HEI-2015**			
Total scores	50.7 ± 14.0	47.9 ± 13.5	< 0.0001
Total vegetables	3.1 ± 1.7	2.8 ± 1.7	< 0.0001
Greens and beans	1.7 ± 2.2	1.3 ± 2.0	< 0.0001
Total fruits	1.9 ± 2.0	1.7 ± 2.0	0.0052
Whole fruits	2.1 ± 2.3	1.8 ± 2.2	0.0033
Whole grains	2.4 ± 3.4	2.2 ± 3.2	0.0618
Dairy	4.8 ± 3.4	4.7 ± 3.3	0.1808
Total protein foods	4.2 ± 1.3	4.2 ± 1.3	0.2627
Seafood and plant	2.4 ± 2.3	2.3 ± 2.3	0.3515
Fatty acids	5.0 ± 3.6	4.6 ± 3.7	0.0011
Sodium	4.6 ± 3.5	4.5 ± 3.5	0.4287
Refined grains	6.4 ± 3.7	5.9 ± 3.7	0.0002
Saturated fats	5.4 ± 3.5	5.1 ± 3.5	0.0328
Added sugars	6.9 ± 3.4	6.7 ± 3.4	0.0251

BMI, body mass index; PIR, poverty income ratio; eGFR, estimated glomerular filtration rate; ALT, alanine transaminase; AST, aspartate transaminase; ALP, alkaline phosphatase; LDH, lactate dehydrogenase; HbA1c, glycated hemoglobin; HDL, high density lipoprotein; LDL, low density lipoprotein; hsCRP, high sensitivity C-reactive protein; HEI, healthy eating index; SD standard deviation; %, weighted percentage.

### 3.2 Associations of HEI-2015 with MAFLD

#### 3.2.1 Total analyses

**Figure 2:**
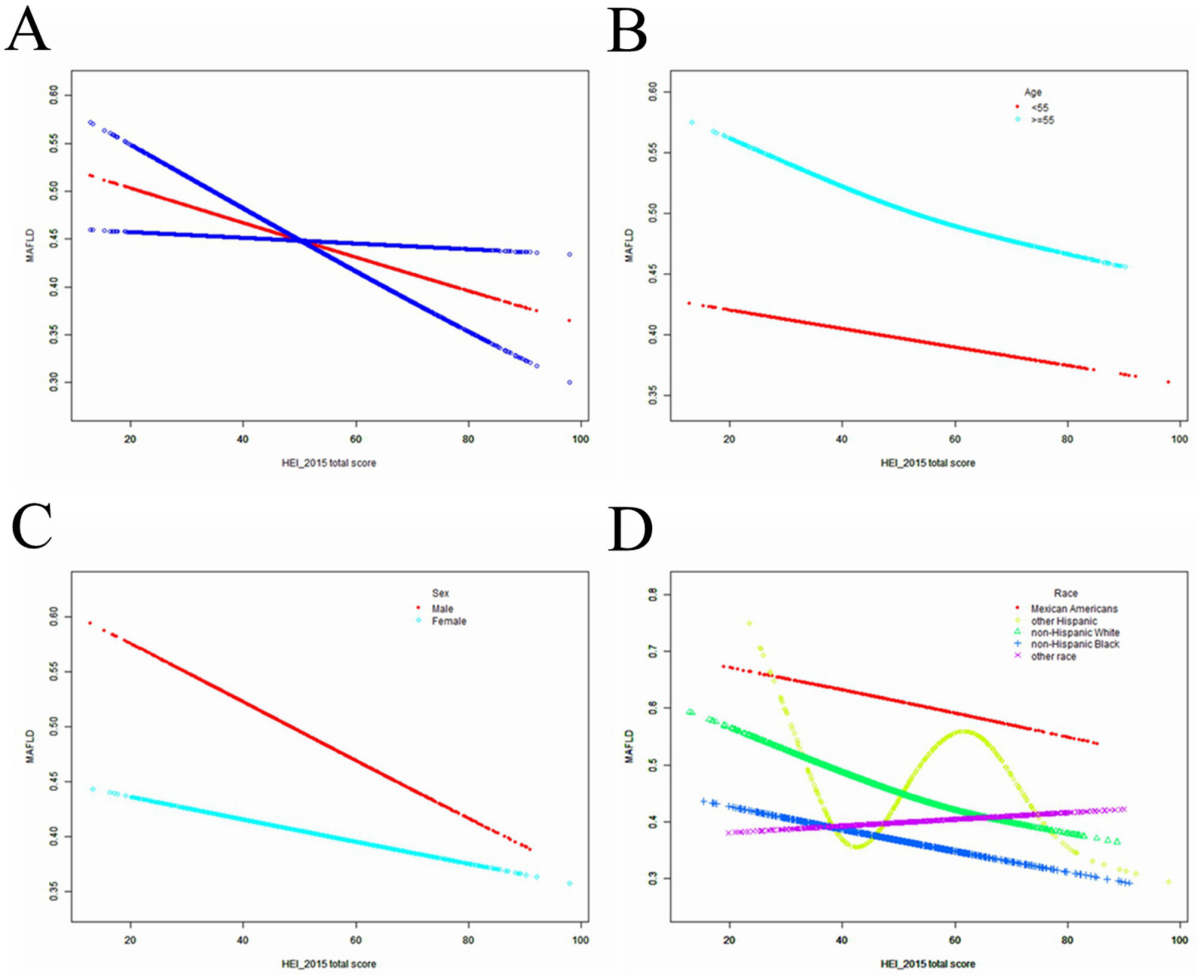
The SCF for associations of HEI-2015 total score with osteoporosis. **(A)** Represents the overall trend. **(B–D)** Represent the substratum trends grouped by age, gender and race, respectively. Age (if applicable), sex (if applicable), race, BMI, PIR, educational level, marital status, smoked at least 100 cigarettes, hypertension status, diabetes status, eGFR, moderate or vigorous activity, alcohol consumption, AST/ALT, Albumin/Globulin, GGT, ALP, LDH, Total bilirubin, Uric acid, HbA1c, Total cholesterol and hsCRP were adjusted.

HEI-2015 total/component scores showed a negative association with MAFLD in Model 1. However, after adjusting for confounding factors in Models 2 (age, sex, and race) and 3 (age, sex, race, race, BMI, PIR, educational level, marital status, smoked at least 100 cigarettes, hypertension status, diabetes status, eGFR, moderate or vigorous activity, alcohol consumption, AST/ALT, Albumin/Globulin, GGT, ALP, LDH, Total bilirubin, Uric acid, HbA1c, Total cholesterol and hsCRP, the relationship between exposed variables and outcomes remained stable (Table 2). When adjusting for all covariates, each unit of increased HEI-2015 value was associated with a decreased risk of MAFLD of 1.2% (Table 2). Furthermore, after adjusting for all covariates, the negative associations between HEI-2015 and MAFLD were also observed in smooth curve fitting (SCF) and weighted generalized additive model (GAM; Figure 2A).

**Table 2:** Association of the HEI-2015 and the risk of MAFLD.

	**Male β (95%CI) *P* value**	**Female β (95%CI) *P* value**	**Total β (95%CI) *P* value**
**Age**<**55**			
Model 1	0.982 (0.978, 0.986)	0.977 (0.973, 0.981)	0.979 (0.976, 0.982)
Model 2	0.980 (0.976, 0.985)	0.976 (0.972, 0.980)	0.978 (0.975, 0.981)
Model 3	0.977 (0.970, 0.983)	0.985 (0.979, 0.991)	0.983 (0.979, 0.988)
**Age** ≥**55**			
Model 1	0.997 (0.992, 1.001)^*^	0.976 (0.971, 0.980)	0.986 (0.983, 0.989)
Model 2	0.996 (0.991, 1.001)^*^	0.974 (0.969, 0.979)	0.984 (0.981, 0.987)
Model 3	1.005 (0.998, 1.012)^*^	0.980 (0.975, 0.986)	0.989 (0.985, 0.994)
**Total**			
Model 1	0.988 (0.985, 0.992)	0.977 (0.974, 0.980)	0.982 (0.980, 0.985)
Model 2	0.987 (0.984, 0.990)	0.975 (0.972, 0.978)	0.981 (0.979, 0.983)
Model 3	0.990 (0.986, 0.995)	0.984 (0.980, 0.988)	0.988 (0.985, 0.991)

Model 1: no covariates were adjusted.

Model 2: age (if applicable), sex (if applicable), and race were adjusted.

Model 3: age (if applicable), sex (if applicable), race, BMI, PIR, educational level, marital status, smoked at least 100 cigarettes, hypertension status, diabetes status, eGFR, moderate or vigorous activity, alcohol consumption, AST/ALT, Albumin/Globulin, GGT, ALP, LDH, Total bilirubin, Uric acid, HbA1c, Total cholesterol and hsCRP were adjusted.

^*^Indicates P > 0.05.

After classifying the HEI-2015 based to quartiles, the OR between the risk of MAFLD and value of HEI-2015 for quintiles 2, 3, and 4 compared with quintile 1 was 0.945 (95% CI: 0.852, 1.047; *P*= 0.27972), 0.834 (95% CI: 0.750, 0.927; *P* < 0.000001) and 0.723 (95% CI: 0.646, 0.811; *P* < 0.000001), respectively, in Model 3. The trend test also showed that the risk of MAFLD decreased as the HEI-2015 quartile group increased (for trend, *P* < 0.001)

#### 3.2.2 Subgroup analyses

After stratifying the participants by age or sex, the subgroup analyses presented a similar trend to the above. Between Model 1, Model 2, or Model 3, MAFLD and HEI-2015 were negatively correlated in subgroup analyses by age or sex. After adjusting for all covariates, the negative associations between HEI-2015 and MAFLD were also observed in smooth curve fitting (SCF) and weighted generalized additive model (GAM; Figures 2B, C). Moreover, race-stratified subgroup analyses confirmed the negative correlation between MAFLD and HEI-2015 among Mexican Americans, Non-Hispanic White people and Non-Hispanic Black people, as shown in SCF and GAM (Figure 2D). When adjusting for all covariates except age, each unit of increased HEI-2015 total score was associated with 1.7% (95% CI: 1.2%, 2.1%; *P* < 0.00001) and 1.1% (95% CI: 0.6%, 1.5%; *P* < 0.00001) decreased risk of MAFLD in people aged below 55 years and those aged 55 years or older, respectively (Table 2).

Moreover, in people aged below 55 years, the ORs between the risk of MAFLD and HEI-2015 total score across quintiles 2, 3, and 4 compared with quintile 1 were 0.861 (95% CI: 0.752, 0.986; *P* = 0.03029), 0.858 (95% CI: 0.745, 0.989; *P* = 0.03404), and 0.698 (95% CI: 0.593, 0.822; *P* = 0.00002), respectively (Table 3). In people who were 55 years or older, the ORs were 0.973 (95% CI: 0.823, 1.151; *P* = 0.75270), 0.774 (95% CI: 0.653, 0.917; *P* = 0.00306), and 0.679 (95% CI: 0.573, 0.804; *P* < 0.000001), respectively (Table 3). When adjusting for all covariates except sex, each unit of increased HEI-2015 total score was associated with 1.0% (95% CI: 0.5%, 1.4%; *P* < 0.00001) and 1.6% (95% CI: 1.2%, 2.0%; *P* < 0.00001) decreased risk of MAFLD in male and female (Table 2).

**Table 3:** Associations of the HEI-2015.Q4 with risk of MAFLD.

	**Male (95% CI) *P* value**	**Female (95% CI) *P* value**	**Total β (95% CI) *P* value**
**Age**<**55**			
**Model 1**			
Q1	1	1	1
Q2	0.747 (0.647, 0.862)	1.119 (0.962, 1.302)^*^	0.909 (0.819, 1.008)^*^
Q3	0.898 (0.775, 1.042)^*^	0.580 (0.496, 0.680)	0.721 (0.648, 0.802)
Q4	0.505 (0.425, 0.601)	0.568 (0.480, 0.673)	0.537 (0.476, 0.606)
**Model 2**			
Q1	1	1	1
Q2	0.715 (0.618, 0.827)	1.104 (0.948, 1.285)^*^	0.885 (0.797, 0.983)
Q3	0.870 (0.747, 1.012)^*^	0.581 (0.495, 0.681)	0.711 (0.638, 0.793)
Q4	0.493 (0.413, 0.588)	0.537 (0.453, 0.638)	0.511 (0.452, 0.577)
**Model 3**			
Q1	1	1	1
Q2	0.564 (0.460, 0.692)	1.395 (1.139, 1.709)	0.861 (0.752, 0.986)
Q3	0.736 (0.596, 0.908)	0.882 (0.709, 1.097)^*^	0.858 (0.745, 0.989)
Q4	0.581 (0.451, 0.749)	0.796 (0.630, 1.006)^*^	0.698 (0.593, 0.822)
*P* trend	< 0.001	< 0.001	< 0.001
**Age** ≥**55**			
**Model 1**			
Q1	1	1	1
Q2	0.907 (0.745, 1.104)^*^	0.864 (0.712, 1.048)^*^	0.887 (0.772, 1.018)^*^
Q3	0.711 (0.584, 0.865)	0.853 (0.707, 1.029)^*^	0.794 (0.693, 0.909)
Q4	0.842 (0.691, 1.026)^*^	0.436 (0.361, 0.526)	0.586 (0.512, 0.671)
**Model 2**			
Q1	1	1	1
Q2	0.903 (0.741, 1.101)^*^	0.855 (0.704, 1.039)^*^	0.870 (0.757, 1.000)
Q3	0.691 (0.567, 0.843)	0.809 (0.669, 0.978)	0.757 (0.660, 0.869)
Q4	0.816 (0.667, 0.997)	0.415 (0.343, 0.502)	0.555 (0.484, 0.636)
**Model 3**			
Q1	1	1	1
Q2	0.942 (0.731, 1.213)^*^	1.015 (0.799, 1.289)^*^	0.973 (0.823, 1.151)^*^
Q3	0.675 (0.517, 0.881)	0.845 (0.667, 1.070)^*^	0.774 (0.653, 0.917)
Q4	1.113 (0.842, 1.470)^*^	0.535 (0.423, 0.677)	0.679 (0.573, 0.804)
*P* trend	0.019	< 0.001	< 0.001
**Total**			
**Model 1**			
Q1	1	1	1
Q2	0.798 (0.711, 0.896)	1.004 (0.891, 1.131)^*^	0.896 (0.825, 0.974)
Q3	0.801 (0.711, 0.902)	0.705 (0.626, 0.793)	0.749 (0.689, 0.815)
Q4	0.650 (0.573, 0.737)	0.491 (0.434, 0.556)	0.559 (0.512, 0.611)
**Model 2**			
Q1	1	1	1
Q2	0.780 (0.694, 0.876)	0.987 (0.875, 1.113)^*^	0.877 (0.806, 0.953)
Q3	0.775 (0.687, 0.874)	0.691 (0.613, 0.780)	0.729 (0.670, 0.794)
Q4	0.627 (0.552, 0.714)	0.468 (0.413, 0.531)	0.532 (0.486, 0.582)
**Model 3**			
Q1	1	1	1
Q2	0.743 (0.639, 0.864)	1.203 (1.037, 1.395)	0.945 (0.852, 1.047)^*^
Q3	0.689 (0.589, 0.807)	0.919 (0.789, 1.070)^*^	0.834 (0.750, 0.927)
Q4	0.771 (0.646, 0.920)	0.674 (0.575, 0.790)	0.723 (0.646, 0.811)
*P* trend	< 0.001	< 0.001	< 0.001

Model 1: no covariates were adjusted.

Model 2: age (if applicable), sex (if applicable), and race were adjusted.

Model 3: age (if applicable), sex (if applicable), race, BMI, PIR, educational level, marital status, smoked at least 100 cigarettes, hypertension status, diabetes status, eGFR, moderate or vigorous activity, alcohol consumption, AST/ALT, Albumin/Globulin, GGT, ALP, LDH, Total bilirubin, Uric acid, HbA1c, Total cholesterol and hsCRP were adjusted.

Q1: 12.8252–39.9288, Q2: 39.9296–49.3031, Q3: 49.3072–59.9345, Q4: 59.9582–97.8766.

^*^Indicates P > 0.05.

In male, the ORs between the risk of MAFLD and HEI-2015 total score across quintiles 2, 3, and 4 compared with quintile 1 were 0.743 (95% CI: 0.639, 0.864; *P* =0.00012), 0.689 (95% CI: 0.589, 0.807 *P* < 0.00001), and 0.771 (95% CI: 0.646, 0.920; *P* = 0.00385), respectively (Table 3). In female, the ORs were 1.203 (95% CI: 1.037, 1.395; *P* = 0.01451), 0.919 (95% CI: 0.789, 1.070, *P*= 0.27728), and 0.674 (95% CI: 0.575, 0.790; *P* < 0.00001), respectively (Table 3). Regardless of whether men or women were over 55 years of age, a trend test showed that the risk of MAFLD decreased with increasing HEI-2015 quartile group (trend test, *P* < 0.001; Table 3).

After further cross-stratifying the participants by age and gender, it was found that HEI-2015 was negatively associated with MAFLD except for men aged of 55 years or older (Table 2). Then, subgroup analyses then showed that there was no significant association between HEI-2015 and MAFLD in men aged 55 years and older in Models 1, 2, and 3. In male aged 55 years old or older, the ORs between the risk of MAFLD and HEI-2015 total score across quintiles 2, 3, and 4 compared with quintile 1 were 0.942 (95% CI: 0.731, 1.213; *P* = 0.64204),0.675 (95% CI: 0.517, 0.881 *P* = 0.00375), and 1.113 (95% CI:0.842, 1.470; *P* = 0.45194), respectively (Table 3).

## 4 Discussion

Based on a representative sample from the National Health and Nutrition Examination Survey (NHANES, 2017-2018), the investigation revealed a significant negative correlation between the Healthy Eating Index 2015 (HEI-2015) scores and the risk of Metabolic Associated Fatty Liver Disease (MAFLD) among participants. Furthermore, the analysis indicated that these associations remained consistent across various age and sex subgroups. The results imply that adherence to the recommended consumption levels of the 13 food components outlined in the HEI-2015, specifically total vegetables, greens and beans, total fruits, whole fruits, fatty acids, and refined grains, is correlated with a reduced risk of MAFLD.

MAFLD is a complex condition characterized by obesity, diabetes, and a constellation of metabolic disorders (13). This disease exhibit significant metabolic derangements and hepatic damage, as evidenced by increased levels of liver enzymes such as ALT, AST, GGT, and ALP, as well as HbA1c, LDL, and triglycerides. Our findings indicate that a higher proportion of individuals within the MAFLD cohort exhibit these metabolic abnormalities and hepatic dysfunctions, which are consistent with the characteristic features of MAFLD.

After additional adjustments for confounding factors, the persistent preventive and protective effects of diet on MAFLD were observed, suggesting that dietary factors are crucial in the prevention and control of MAFLD, unaffected by other influences. This negative correlation is consistent across age and gender subgroups, suggesting that the protective effect of a high-quality diet on MAFLD is ubiquitous and not confined to particular demographic segments.

Despite the overall consistency of our results, we observed no significant negative correlation between HEI-2015 scores and MAFLD in males aged 55 and above. This may be attributed to additional confounding factors peculiar to this demographic cohort, including adiposity distribution, hormonal status, ethnicity, dietary habits, alcohol consumption, smoking, genetic predisposition, gut microbiota, and metabolic profile, which may modulate the relationship between diet quality and MAFLD (15–18).

Why does a low-quality diet contribute to the development of MAFLD? Notably, an elevated intake of fat and fructose contributes significantly to the increase in obesity and fatty liver disease, which are conditions associated with various metabolic dysfunctions, including insulin resistance and dyslipidemia (19). Furthermore, excessive fructose consumption correlates with increased fat deposition and elevated hepatic mRNA expression of fructokinase and fatty acid synthase. Fructose also exacerbates oxidative stress by reducing antioxidant defenses and increasing the generation of reactive oxygen species (ROS), which may lead to necroinflammation (20). These dietary components are indicative of the Western dietary pattern, which is rich in protein sources such as cheese, processed meats, pastries, pizza, chips, snack foods, and refined grains (21). This dietary pattern starkly contrasts with the guidelines set forth by HEI-2015. We observed that patients with MAFLD did not have low scores in categories such as Whole Grains, Dairy, Total Protein Foods, Seafood, and Plant-based foods. This finding is consistent with the characteristics of a Western diet.

In contrast to Western dietary, the Mediterranean dietary pattern is recognized as a high-quality dietary pattern. Numerous studies have evidenced that the Mediterranean dietary pattern can prevent or even reverse MAFLD (6, 7, 22). The Mediterranean diet is distinguished by a high consumption of olive oil, vegetables, fruits, whole grains, nuts, and legumes; a moderate intake of fish, lean meats, dairy products, and red wine; and a low consumption of eggs and sweets (23). Our study demonstrated that individuals with MAFLD had a significantly lower intake of total vegetables, greens and legumes, total and whole fruits, fatty acids, and refined grains compared to the non-MAFLD cohort. These food components conform to the recommendations of the Mediterranean dietary pattern. Prior research has established a positive correlation between HEI-2015 and the Mediterranean Diet (MED) score (24). This result provides additional support for the hypothesis that higher HEI-2015 scores are associated with a lower risk of MAFLD.

Our study has several strengths. First, we used a large, nationally representative database to estimate dietary quality, adhered to strict control procedures, and used the most recent version of the HEI index for analyses. Second, we adequately controlled for confounders in our study and conducted HEI-2015 component analyses to further explore the association between HEI-2015 scores and MAFLD. There are some potential limitations to our analyses. We were unable to determine causality because our study was a cross-sectional analysis. Second, whereas dietary data were collected based on two 24-h retrospectives at two time points in the cross-sectional survey design, this does not reflect usual nutrient intake, which may have affected the results. In addition, we were unable to include all potentially meaningful variables due to database data.

## 5 Conclusion

Elevated dietary quality, as assessed by the total and component food scores of the HEI-2015, was significantly correlated with a diminished risk of MAFLD among participants in the NHANES survey featured in this investigation.

## Data Availability

Publicly available datasets were analyzed in this study. The NHANES website (accessed on 15 March 2024) provides access to the datasets that were used and/or analyzed during the current investigation.
